# Visually Guided Avoidance in the Chameleon (*Chamaeleo chameleon*): Response Patterns and Lateralization

**DOI:** 10.1371/journal.pone.0037875

**Published:** 2012-06-07

**Authors:** Avichai Lustig, Hadas Ketter-Katz, Gadi Katzir

**Affiliations:** 1 Department of Neurobiology and Ethology, University of Haifa, Haifa, Israel; 2 Department of Environmental and Evolutionary Biology, University of Haifa, Haifa, Israel; 3 Department of Marine Biology, University of Haifa, Haifa, Israel; 4 Department of Biology, University of Haifa at Oranim, Tivon, Israel; Lund University, Sweden

## Abstract

The common chameleon, *Chamaeleo chameleon,* is an arboreal lizard with highly independent, large-amplitude eye movements. In response to a moving threat, a chameleon on a perch responds with distinct avoidance movements that are expressed in its continuous positioning on the side of the perch distal to the threat. We analyzed body-exposure patterns during threat avoidance for evidence of lateralization, that is, asymmetry at the functional/behavioral levels. Chameleons were exposed to a threat approaching horizontally from the left or right, as they held onto a vertical pole that was either wider or narrower than the width of their head, providing, respectively, monocular or binocular viewing of the threat. We found two equal-sized sub-groups, each displaying lateralization of motor responses to a given direction of stimulus approach. Such an anti-symmetrical distribution of lateralization in a population may be indicative of situations in which organisms are regularly exposed to crucial stimuli from all spatial directions. This is because a bimodal distribution of responses to threat in a natural population will reduce the spatial advantage of predators.

## Introduction

Changes in body orientation in response to external stimuli are fundamental to animal motion and locomotion and require the perception of one’s location in relation to the relevant stimuli. Frequent examples are provided by visually guided responses, including cases in which animals perform highly accurate spatio-temporal corrections of their body or organ position relative to a moving stimulus. Such position corrections, often referred to as “station keeping” [1]–[5], are observed, for example, in bees maintaining position in front of their hives, or hoverflies closely tracking females in courtship [1]–[6]. Body position corrections are often observed in avoidance, such as in the case of locusts (*Locusta migratoria*). When holding onto a twig or branch and exposed to a threat, a locust will respond by actively positioning itself so as to keep on the far side of its perch. Such behavior is performed only while the threat is in motion, resulting in minimizing its exposure to that threat [7]. Similar behavior patterns are observed in grasshoppers and cicadas and may well reduce the chances of detection.

Chameleons (Chamaeleonidae, Reptilia) are slow-moving, predominantly arboreal lizards that capture insect prey with a long tongue. Chameleons rely on cryptic coloration and slow motion to approach prey and to reduce visual detection by potential predators [8], [9]. Their concealment or evasive motor patterns are related to the level of threat, with a low-level, distant threat more likely to elicit “freezing” of slow motion and a high-level, nearby threat more likely to elicit “free-falling” escape or gaping behavior. In the “free fall” response, the chameleon suddenly drops from its perch to the shrubs below, and with the use of cryptic color change attempts an escape.

When a threat appears on the side of a branch opposite to that on which a chameleon is perched, the chameleon will often remain motionless [8], [9]. However, if the threat appears at other angles, and is more fully exposed to the chameleon, the chameleon will flatten its torso bilaterally and rotate on the branch so that its ventral side, in the direction of the threat, is minimally exposed (Lustig et al., unpublished data). Throughout, the chameleon visually tracks the threat and adjusts its body position with smooth rotational motions, even if the threat is several meters away.

In this avoidance response, as with other motor responses of animals with bilateral morphological symmetry, the question of laterality arises. Is there an effect of direction of the stimulus’ approach on the motor patterns displayed by the chameleon? While the spatio-temporal patterns of eye use have been analyzed [10], the movements of the chameleon’s body as it attempts to hide from the threat remain unstudied.

Bilateral symmetry in vertebrates is widely expressed morphologically and anatomically [11], [12]. In the central nervous system, bilateral morphological similarities are observed in the brain and cranial and spinal nerves. However, bilateral similarity in gross anatomy does not necessarily imply bilateral similarity in the neural architecture or consequent behavioral patterns. Lateralization [13] refers to a situation in which the two sides of the body differ in structure, function or both. Lateralization is known to occur in all vertebrate classes [14]–[16], as well as in certain invertebrates with lateralized behavior patterns, and is expressed frequently during foraging, sexual displays, aggression and avoidance responses [14], [17], [18].

Lateralization of motor functions has been described in all poikilotherm groups–fish, amphibians and reptiles. For example, in the mosquitofish (*Gambusia holbrooki*) and *Girardinus falcatus*, body turning during predator evasion is lateralized, while in the zebrafish (*Brachydanio rerio*), exploratory biting of objects is correlated with the use of the right eye [19]–[21]. In the mosquitofish, the red tailed goodeid (*Xenotoca esieni*), and the Siamese fighting fish (*Betta splendens*), visually guided aggressive behavior patterns are elicited using the right eye [22]. In the shiner perch (*Cymatogaster aggregate*), lateralized individuals are faster in their escape responses as compared to unlateralized individuals [23].

**Figure 1:**
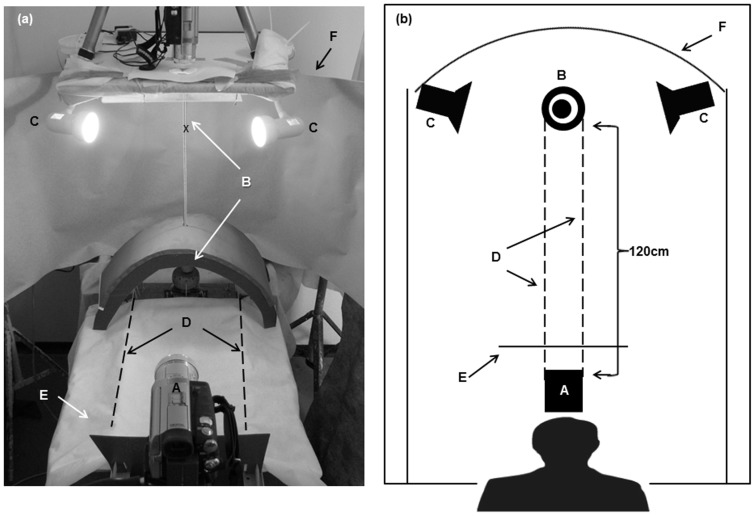
Experimental setup. (A) Oblique view. (B) Schematic overhead view. The experimenter, positioned behind the camera (a) acts as the threatening stimulus. Chameleon (x), vertical pole (b), incandescent bulbs (c), pole rotation cords (d), visual barrier (e), screen (f).

**Figure 2:**
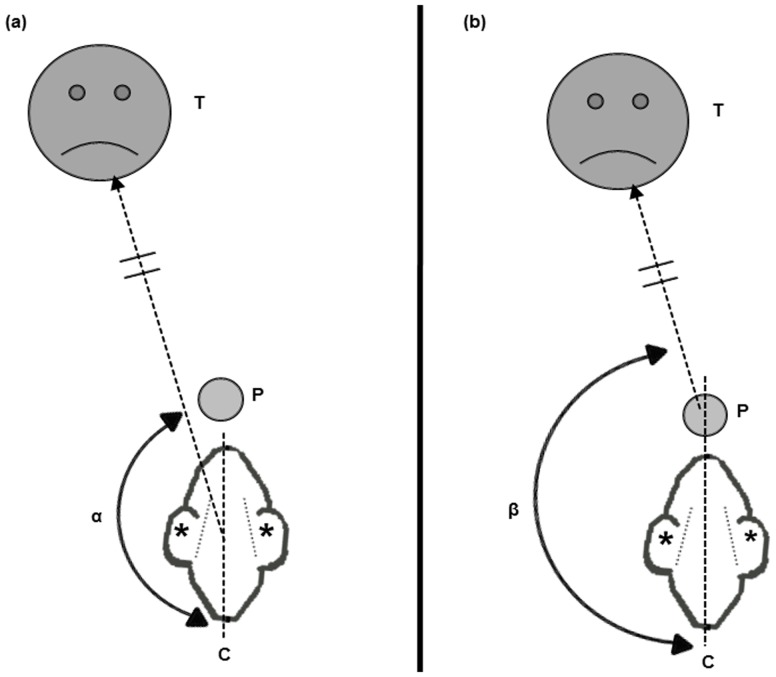
Head angles measured relative to the threat. An overhead view of the sagittal plane of the head of a chameleon (C) when perched vertically on a pole (P), in relation to the threat (T); α – the angle in relation to the threat, β – the angle in relation to both threat and pole.

In amphibians, lateralized motor patterns are found in limb use in toads (e.g., *Bufo bufo, B. marinus* and *B. viridis*) [24], [25]. In the frog *Litoria caerulea*, the right forearm is used mostly in upright-ward motion [26]. Side-dependent predatory behavior and predator-avoidance patterns have been observed in *B. bufo* and *B. viridis* [27], [28].

Among reptiles, lateralized visuo-motor behavior has been found in the aggressive behavior of *Orusaurus ornatus* [29] and *Sceloporus virgatus*, where aggressive charges by females during courtship rejection are more often from the left side [30]. Right-biased predatory responses have been documented in the ornate dragon lizard (*Ctenophorus ornatus*) [31] and in the common wall lizard (*Podarcis muralis*) [32].

Several theories have addressed the possible functions of lateralization. One line of reasoning is that in animals with laterally placed eyes, a feature common to most vertebrates, lateralization reduces inter-hemispheric conflicts. Such conflicts may arise when two stimuli are perceived simultaneously, one by each eye [16], [33]. Hemispheric dominance in a given task, especially under full decussation of the optic nerves, may be a means of reducing or eliminating the dilemma of which stimulus should be responded to. Another possible function is related to neural processing. In most examined vertebrates, each hemisphere is more highly specialized in attending to, or responding to certain stimulus categories such as “threat”, “prey”, or “familiar-novel”. This frees precious cognitive “storage space” and reduces redundancy of brain function [16].

Lateralization may occur at the individual level, population level, or both. An equal distribution of lateralization in the population (termed “anti-symmetrical”) implies that approximately one half of the individuals are biased toward one side while the other half is biased toward the opposite side. An asymmetrical distribution occurs when a significant proportion of the population is biased toward a given side [16]. This bias may be expressed in the response to a given visual stimulus, motor function, or morphological/anatomical feature.

In this study we analyze the motor responses of chameleons faced with an approaching threat in an attempt to provide insight into the dynamics of the response and to assess whether it is lateralized at the individual level, population level or both. In their natural habitats, chameleons mostly move in relatively thick, homogeneous vegetation. Threats, such as predators, may appear from any distance or direction with equal probability. Having an avoidance response that is lateralized, i.e., biased toward a given side, may be detrimental to survival. We therefore hypothesize that the avoidance response of the chameleon will not be side-dependent.

**Figure 3:**
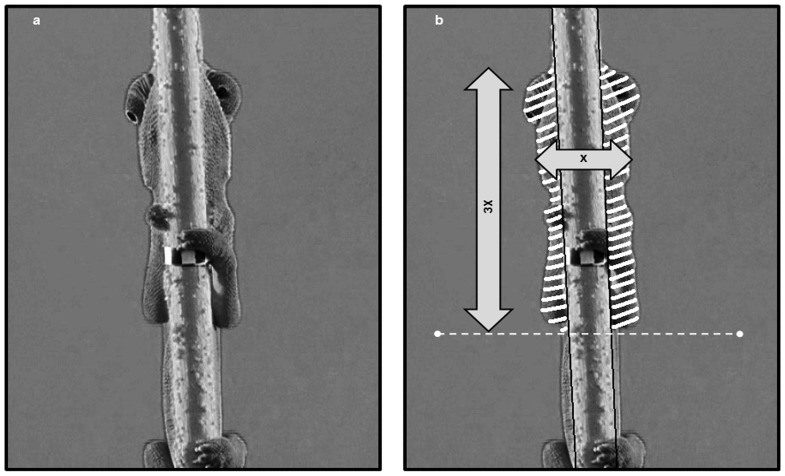
Ventral surface exposure used in the avoidance response analysis. A single frame from a sampled video sequence is depicted. (A) Unmodified image showing the ventral view of the chameleon holding onto a narrow pole, with its eyes protruding from both sides of the pole. (B) Body surface of the chameleon with the areas exposed on each side of the pole (hatched) used for the determination of respective surfaces. The caudal border of the area analyzed (broken horizontal line) is determined on the basis of 3×maximal head width, from the rostral end.

**Figure 4:**
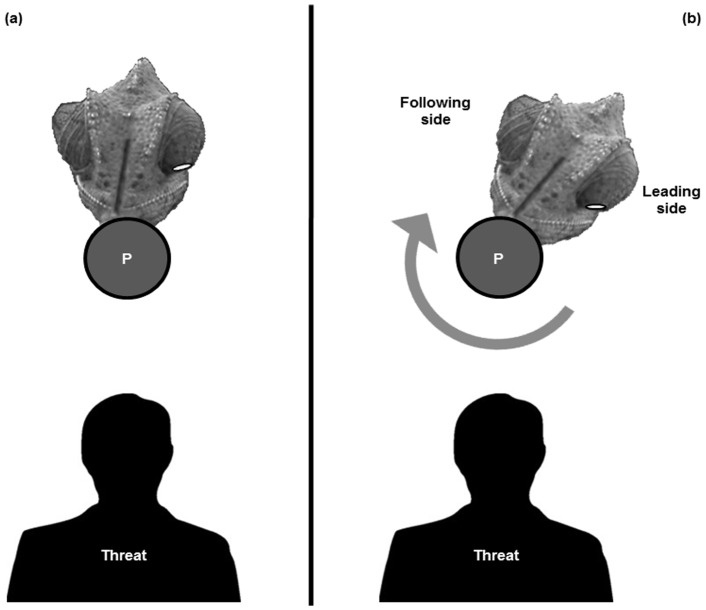
Definitions of body side motion. A chameleon perched on a vertical pole (P) and the threat, as viewed from above. (A) The chameleon is positioned opposite (ca.180°) the threat, in an initial state. (B) The position of the chameleon during, or immediately following, pole rotation. A given side of a chameleon is termed the “leading side” if the threat approaches from that side (i.e., the left side of the chameleon during left-approaching threat, as shown here, or the right side of the chameleon during right-approaching threat). The side opposite the leading side in each test is termed the “following side.”

## Materials and Methods

The research was conducted at the Dept. of Biology, University of Haifa, Oranim Campus in Tivon, Israel, between November 2006 and November 2009. Collection, maintenance, and experimentation with the chameleons were performed under permits from the Israeli Nature and Parks Authority (permit 2011/11411) and the University of Haifa ethics committee. Methods are provided here in brief; further details can be found elsewhere [10].

Each tested chameleon was exposed to a threat that approached it from its left or right side in the following manner: the chameleon was placed on a vertical wooden pole that was between 3 mm and 14 mm in diameter. The pole could be rotated on its long axis either clockwise or counter-clockwise. Once the chameleon had settled, the pole was rotated in a 30° step (at ∼15°/s) in a given direction (Phase 1) and was then left stationary, allowing the chameleon to respond (Phase 2). The two phases were termed a “run” and each test comprised three consecutive runs. The experimenter acted as the “threat”, standing stationary ca. 120 cm from the pole so that the pole’s rotation resulted in relative movement of the chameleon toward the threat. Clockwise rotations resulted in a “left-approaching” threat toward the chameleon, while counter-clockwise rotations resulted in a “right-approaching” threat. The poles were either wide or narrow relative to the ventral width of the head of the tested chameleon. The wider pole allowed the chameleon to view the threat only monocularly at any given moment, whereas the narrower pole allowed the chameleon to view the threat both monocularly and binocularly. Each chameleon was tested once with a left-approaching threat and once with a right-approaching threat. Each test comprised three consecutive runs in the given direction. The tests were video-recorded with the camera positioned in front of the experimenter, 120 cm from the tested chameleon and at its level. From this position, the camera’s view was of the chameleon’s ventral side (Fig. 1).

To determine whether the chameleon’s correction of position is a vestibular-driven compensatory response, we performed two control experiments: (a) the pole was rotated without a visual threat and (b) the threat was rotated, while the pole was kept stationary. In control experiment (a), the vertical pole was placed inside an opaque-white plastic sphere, 35 cm in diameter. The chameleons (n  = 4), when perched on the pole, could view only the pole and its base but no obvious threats. In each test, the pole was rotated in succession 10 times clockwise and 10 times counter-clockwise at an angular velocity of ∼15°/s [see Video S1]. In control experiment (b), a threat stimulus (a head figurine 5×10 cm) was moved 50 cm from the pole in an arc of ca. 80° [see Video S2]. The chameleons (n  = 2) were tested for their response to the threat when perched on the pole and level with the threat. Ten tests were performed at each of three angular velocities (15°, 35° and 70°/s). For each angular velocity, three arbitrary tests were chosen for analysis. Two angles were analyzed: angle α–formed by the sagittal plane of the head relative to the threat, and angle β–formed by the sagittal plane of the head relative to the threat, through the pole. Angle β determined the chameleon’s position behind the pole relative to the threat and may be regarded as a measure of the level of visual concealment (Fig. 2).

**Figure 5:**
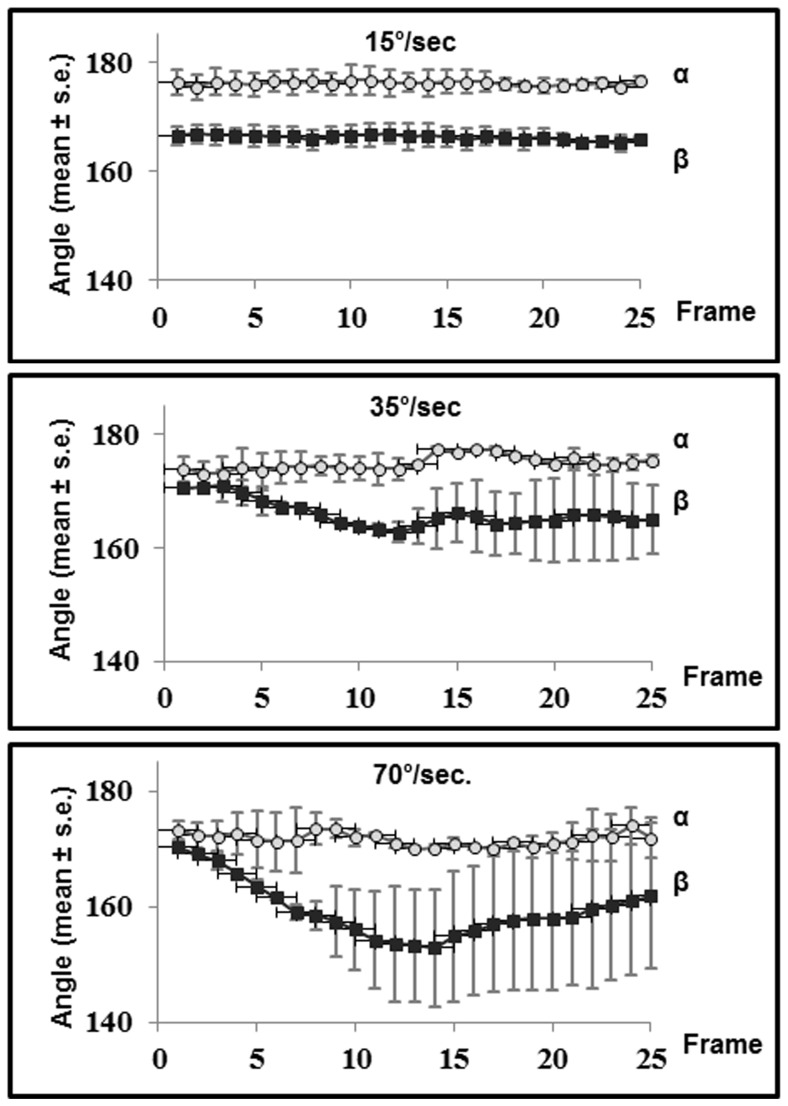
Head angles relative to the moving threat. Provided are the head angles relative to a moving threat under angular velocities of 15°/s, 35°/s and 70°/s. Each data point (mean ± SE) is from six readings (three per chameleon).

**Figure 6:**
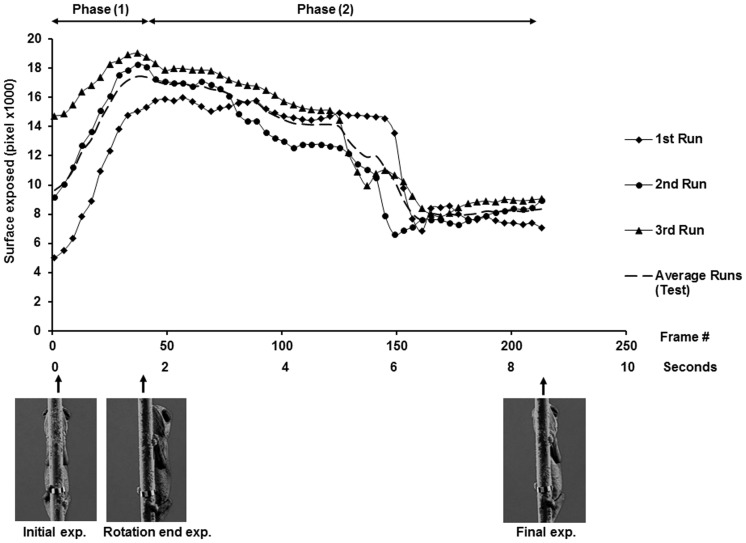
Patterns of motor response of a chameleon on a narrow pole. The degree of body exposure is depicted during three consecutive runs (respectively, triangles, circles, and squares) along with their mean (continuous line). The images are of the chameleon as viewed by the observer (the “threat”) at the respective time points.

### Analysis

Video sequences were edited using Adobe Elements™ software. A specially written program (SIPL Lab, Technion, Israel) sampled the sequences at intervals of four frames (i.e., 160 ms) and provided the size of the surface of the chameleon’s body (in number of pixels) that was exposed on each side of the pole (Fig. 3). To overcome differences in the absolute body size of the tested chameleons and maintain uniformity of the data, a measure of the relative body surface exposed was employed for each chameleon. The maximal ventral width of the head (i.e., mandible width) was measured. Then, from the very rostral end of the head, a distance that was three times the maximal head width was measured caudally and a horizontal line was drawn, forming a caudal “borderline” (Fig. 3).

The tested population comprised chameleons of different sizes, which would have resulted in unequal effects on the statistical tests of the pooled data, since smaller chameleons would have lower exposures by definition. To normalize the data, the tested population was divided into four groups according to head width. Each chameleon in each group had its exposure measurements multiplied by a computed factor which took into account the head width relative to the pole width used in each test. Consequently, data were normalized to the size of the largest chameleons.

The temporal aspect of the response, “latency to final exposure,” was calculated by counting the number of frames from the moment of termination of the pole rotation to the moment (frame) when the chameleon had reached its final exposure and remained still. The data extracted for each sampled frame in each run represented the exposed surface (in pixels) for each chameleon and for each side of the pole, within the above-defined area. In each test, only the side that approached the threat during a given pole rotation, termed the “leading side,” was used for analysis (Fig. 4).

In each exposure of each individual in a given run, three measures were considered: 1) the exposure at the onset of the pole rotation, “Initial exposure”; 2) the exposure at the very end of the rotation, “End of rotation exposure” and 3) the final exposure at the very end of the run, “Final exposure”. Each of the three values was averaged over the three consecutive runs of any given test.

The data were analyzed using repeated measures MANOVA with pole width and direction of threat approach as the main effects. As individuals could be classified as side-biased on a given pole width (see Results), two further analyses were required for each of the biased groups separately.

**Figure 7:**
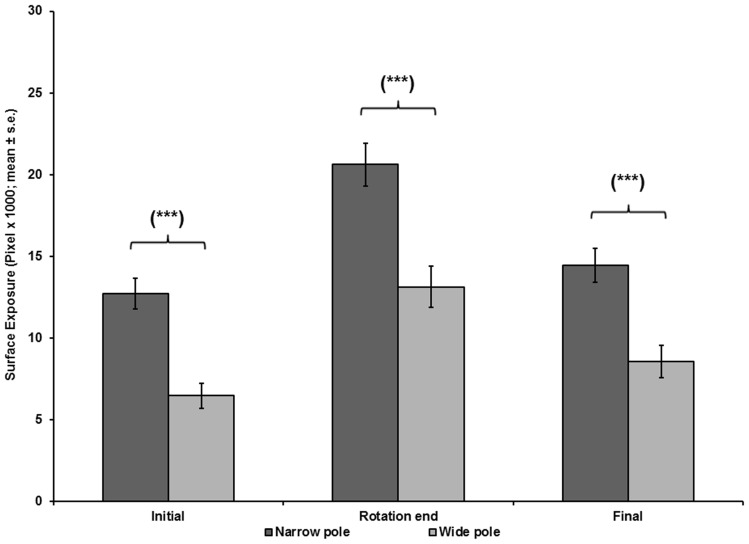
Chameleons’ ventral exposure as a function of pole width. The ventral surface exposed to the threat, on a narrow or wide pole, at the onset of pole rotation (Initial), end of pole rotation (Rotation end), and end of test (Final).

**Figure 8:**
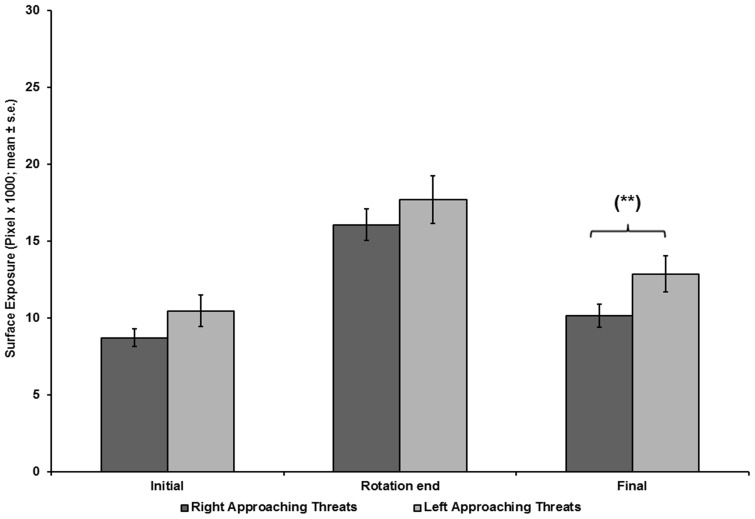
Chameleons’ ventral exposure as a function of threat-approach direction. The ventral surface exposed to a right- or a left-approaching threat at the onset of pole rotation (Initial), end of pole rotation (Rotation end), and end of test (Final).

**Figure 9:**
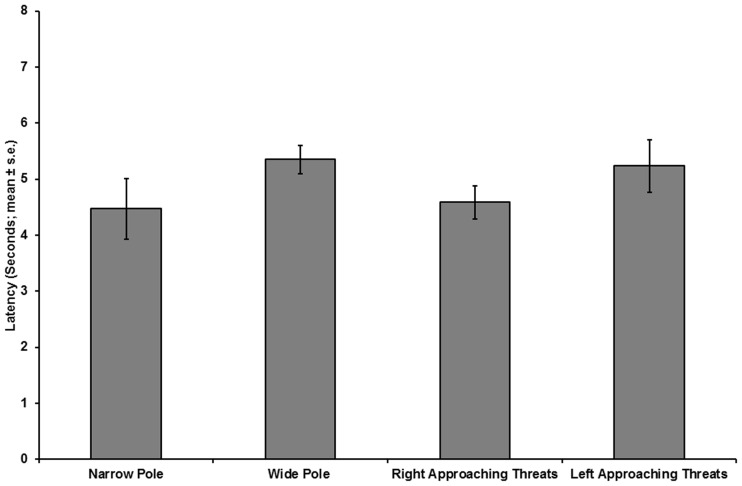
Latency of response as a function of pole width or threat-approach direction. The latency to final exposure on a narrow or wide pole, under a right- or left-approaching threat (N  = 17).

## Results

### Control Experiments

(a) Rotation of the chameleon on the pole within the opaque-white sphere elicited no apparent change of position: the chameleon maintained its position on the pole and rotated with it, clockwise or anti-clockwise [see Video S1]. (b) When perched on a stationary pole and exposed to a threat moving in an arc, the chameleons responded in highly synchronized adjustments of their position relative to the threat (Fig. 5). The analysis showed that both α and β were maintained highly stable in all tests and under all three angular velocities. The angular velocity of 15°/s was the velocity used in the main experiment, as well as in control experiment (a). The results of both control experiments thus demonstrated that the avoidance response of the chameleons is related to the motion of the visual threat and is not elicited by inertia.

### Observed Aspects of the Response

Distinct spatio-temporal motor patterns were observed in the exposure of the chameleon’s body. In Phase 1, an initial increase in ventral body exposure was observed and in Phase 2, there was a decrease in exposure (Fig. 6). Phase 1, which covered the duration from the onset to the termination of pole rotation, resulted in the “leading side” of the chameleon being relatively more exposed to the threat. During Phase 1, exposure at any given moment could be viewed as the product of the rotation itself and the actual movement of the chameleon. Keeping motionless (“frozen”) or moving with the trajectory of the pole would result in increased overall exposure, while moving counter to the rotation of the pole would result in decreased overall exposure. Consequently, a chameleon that actively counter-rotated during pole rotation would reach the end of the perturbation with lower surface exposure than that of a “frozen” chameleon. In Phase 2, the “correction phase,” body exposure changed from the very end of the pole rotation to the end of the test (point of final exposure) due only to the motion of the chameleon in relation to the now stationary threat. This is depicted in Fig. 4, with the decrease in exposure being due to the chameleon’s counter-rotation relative to the threat.

**Figure 10:**
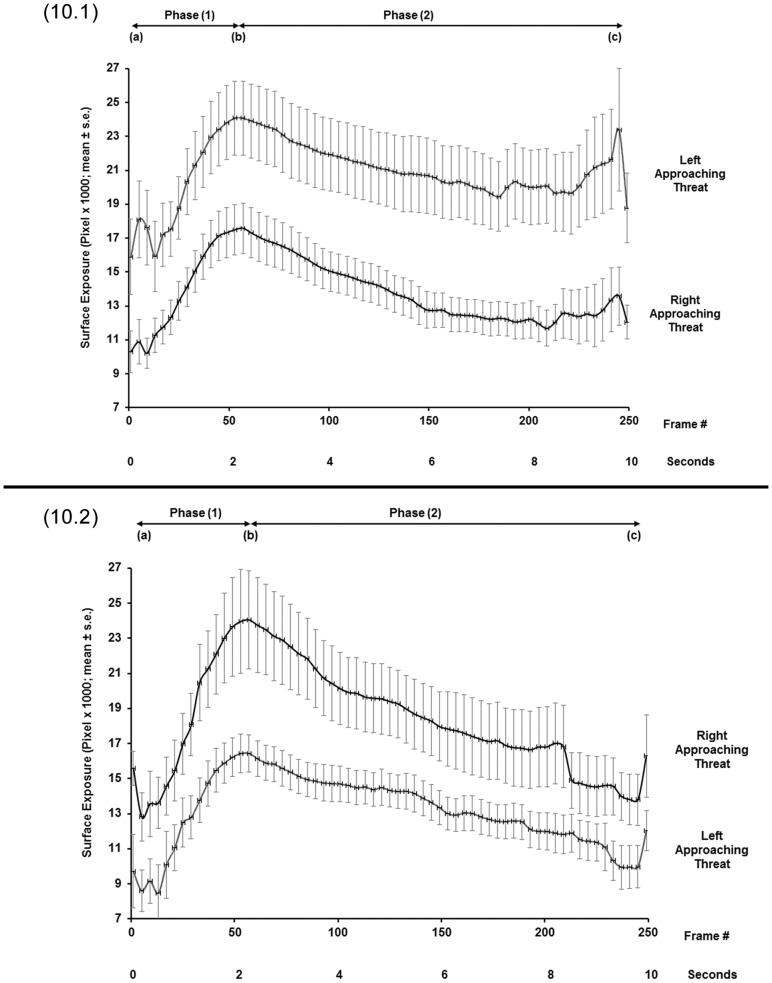
Avoidance response patterns of the two side-biased groups on a narrow pole. Ventral surface exposure (mean ± SE) on narrow poles in response to right- or left-approaching threats in chameleons of the right-biased group (10.1, N  = 14) and of the left-biased group (10.2, N  = 10). Exposure readings are at 200-ms intervals, (A) at the onset of pole rotation, (B) at the end of pole rotation, and (C) at the end of the test.

**Figure 11:**
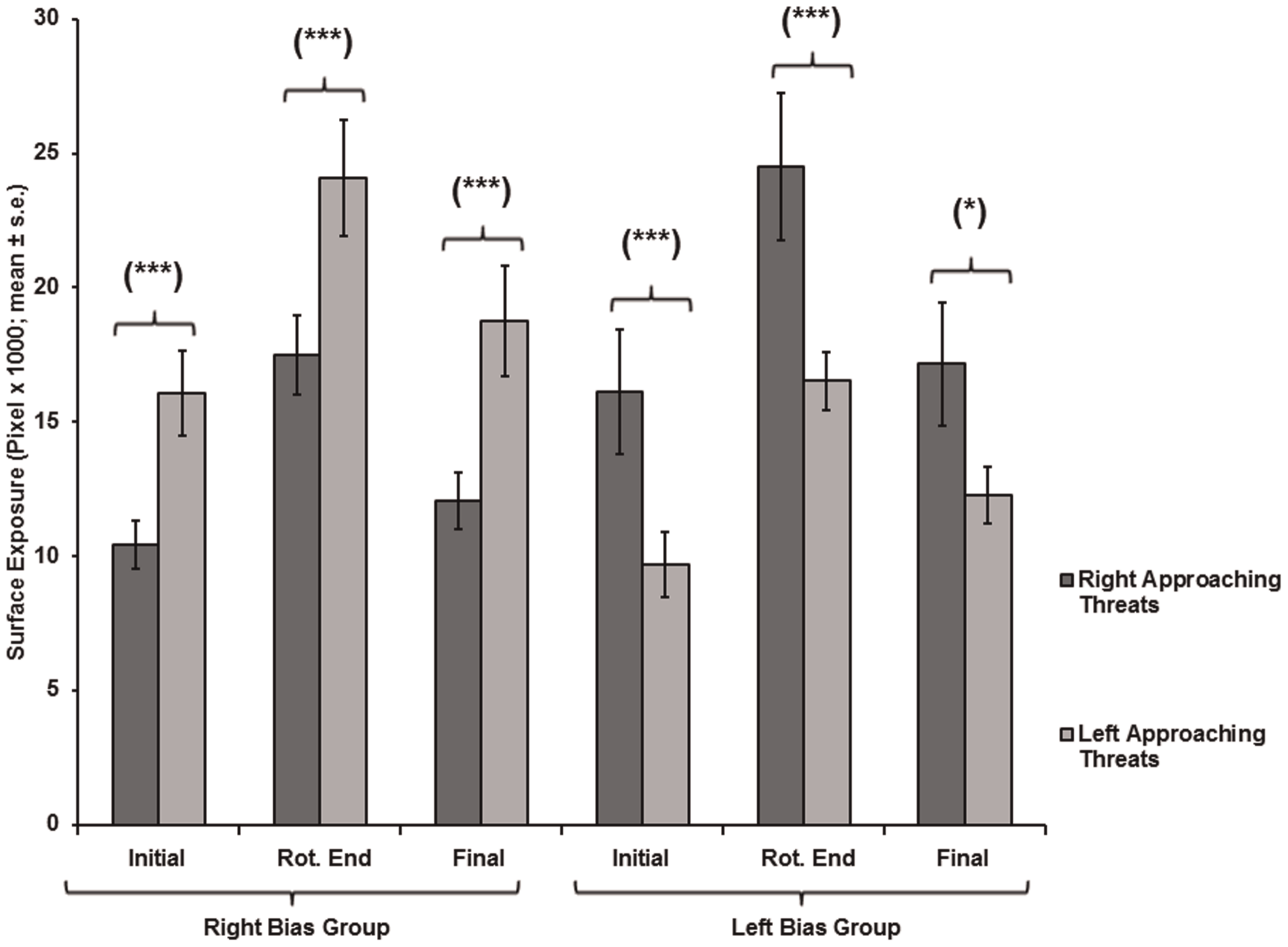
Ventral surface exposure of the two side-biased groups on a narrow pole, as a function of threat-approach direction. Exposure (mean ± SE) during right- or left-approaching threats, for the right-biased (N  = 14) and left-biased (N  = 10) groups, in tests on narrow poles at the onset of pole rotation (Initial), end of pole rotation (Rotation end), and end of test (Final).

**Figure 12:**
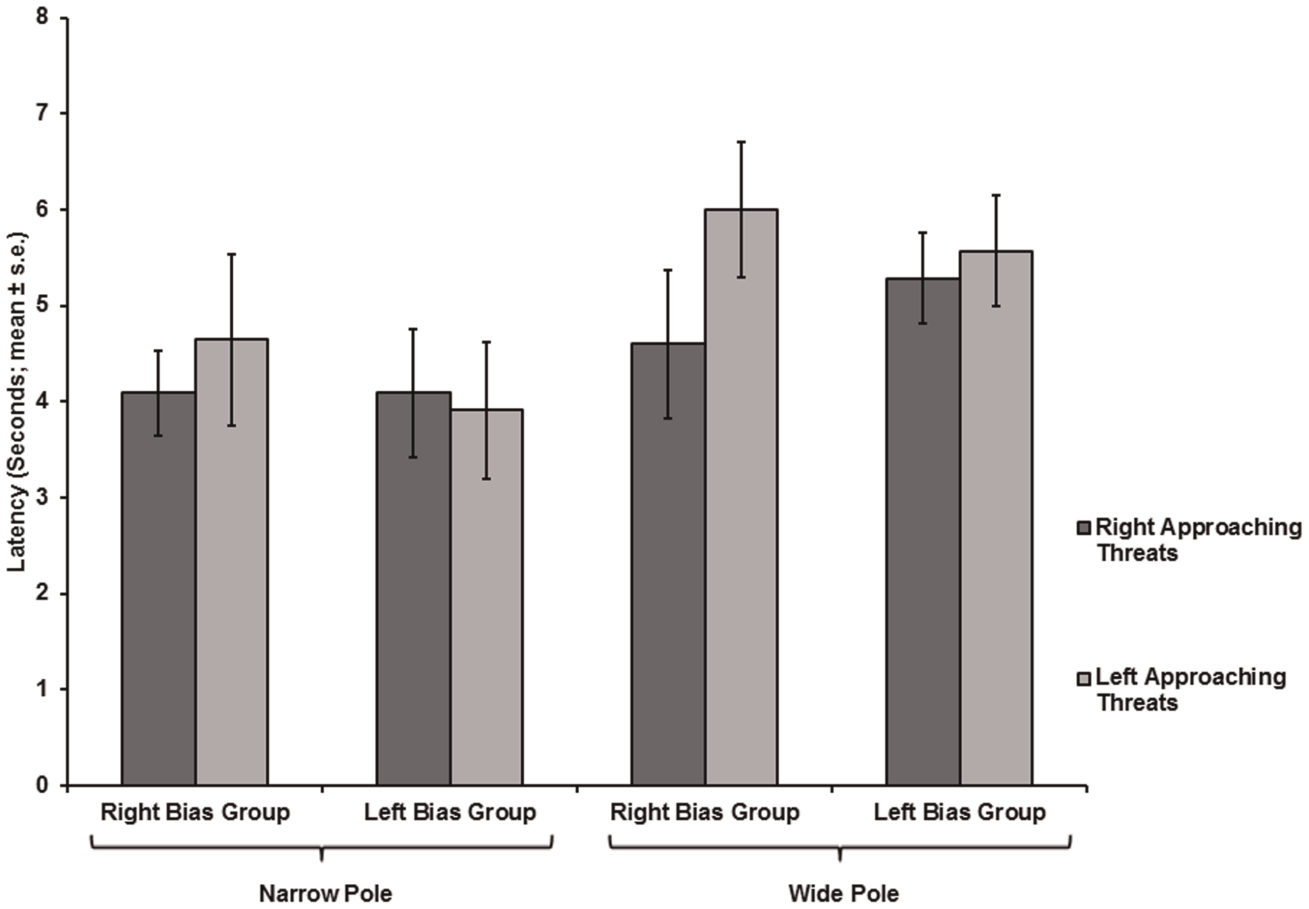
Latency of response of the two side-biased groups as a function of threat-approach direction. Latencies (mean ± SE) to final exposure of chameleons of the right-biased and left-biased groups, under right- or left-approaching threats, on narrow or wide poles (respective number of chameleons tested: 14, 10, 10, 7 for groups from left to right).

A repeated measures MANOVA, with pole width and direction of threat approach as the main effects (Figs. 7, 8), revealed significantly lower exposure in tests on wide poles than in tests on narrow poles, for Initial exposure (F_(1,16)_  = 34.276, p<0.001), End of rotation exposure (F_(1,16)_  = 38.509, p<0.001), and Final exposure (F_(1,16)_  = 25.809, p<0.001). The latency to final exposure in tests on wide poles did not differ from the latency in tests on narrow poles (F_(1,16)_  = 1.704, p  = 0.210). The direction of threat approach did not have a significant effect on the Initial exposure (F_(1,16)_  = 3.39, p  = 0.084) or on the End of rotation exposure (F_(1,16)_  = 1.501, p  = 0.238). The Final exposure during tests with a right-approaching threat was significantly lower than the Final exposure with a left-approaching threat (F_(1,16)_  = 6.233, p  = 0.024). The latency to final exposure (Fig. 9) in tests with right-approaching threats did not differ from the latency in tests with left-approaching threats (F_(1,16)_  = 1.182, p  = 0.293). The interaction between the main effects of pole width and direction of threat approach was not significant (F_(1,16)_  = 0.392, p  = 0.540).

### Within Individual Comparisons

For each chameleon and for a given pole width, a comparison was performed of the mean values of each of the three parameters (i.e., Initial exposure, End of rotation exposure, and Final exposure) between tests of right-approaching threat and left-approaching threat. The means were calculated from the values of a given parameter over the three consecutive runs comprising each test. If two or three of the parameters provided a lower mean value than the parameters for the comparable test on the opposite side, that individual chameleon was considered “side biased.” Of the individuals tested on narrow poles, a proportion of 0.75 were either all higher or all lower in all three spatial parameters than the comparable values in the opposite threat-approach direction. For the wide pole tests, the proportion of individuals was 0.76.

On narrow poles, the proportion of chameleons displaying a bias to right-approaching threats was 0.583 (14/24), whereas the proportion displaying a bias to left-approaching threats was 0.416 (10/24). On wide poles, the proportions were 0.588 (10/17) for the right-side bias and 0.411 (7/17) for the left-side bias. Right-side-biased or left-side-biased individuals were found throughout the tested population in tests on both narrow and wide poles. When examining the entire population, no side bias was observed due to the existence of two sub-groups, each biased toward a given threat-approach direction. Consequently, a further repeated measures MANOVA was performed for each pole width, with the direction of threat approach as a main effect and bias group as a covariate factor. The results showed that, for all three spatial parameters, there was a significant effect of the direction of threat approach (Initial exposure: F_(1,23)_  = 26.273, p<0.001; End of rotation exposure: F_(1,23)_  = 30.437, p<0.001; Final exposure: F_(1,23)_  = 16.486, p<0.001). Moreover, the interaction between the direction of threat approach and the bias group was significant for all three spatial parameters (Initial exposure: F_(1,23)_  = 26.426, p<0.001; End of rotation exposure: F_(1,23)_  = 30.858, p<0.001; Final exposure: F_(1,23)_  = 15.549, p  = 0.001). No differences were found between right- and left-approaching threats in the latency to final exposure, with bias group as a covariate (F_(1,23)_  = 0.58, p  = 0.454). Similarly, the interaction between direction of threat approach and bias group was not significant (F_(1,23)_  = 0.503, p  = 0.485).

Because the interaction between threat-approach direction and bias group was significant with respect to the three spatial parameters, a separate repeated measures MANOVA was conducted for each of the bias groups (i.e., left and right) and for each pole width (i.e., narrow and wide).

**Figure 13:**
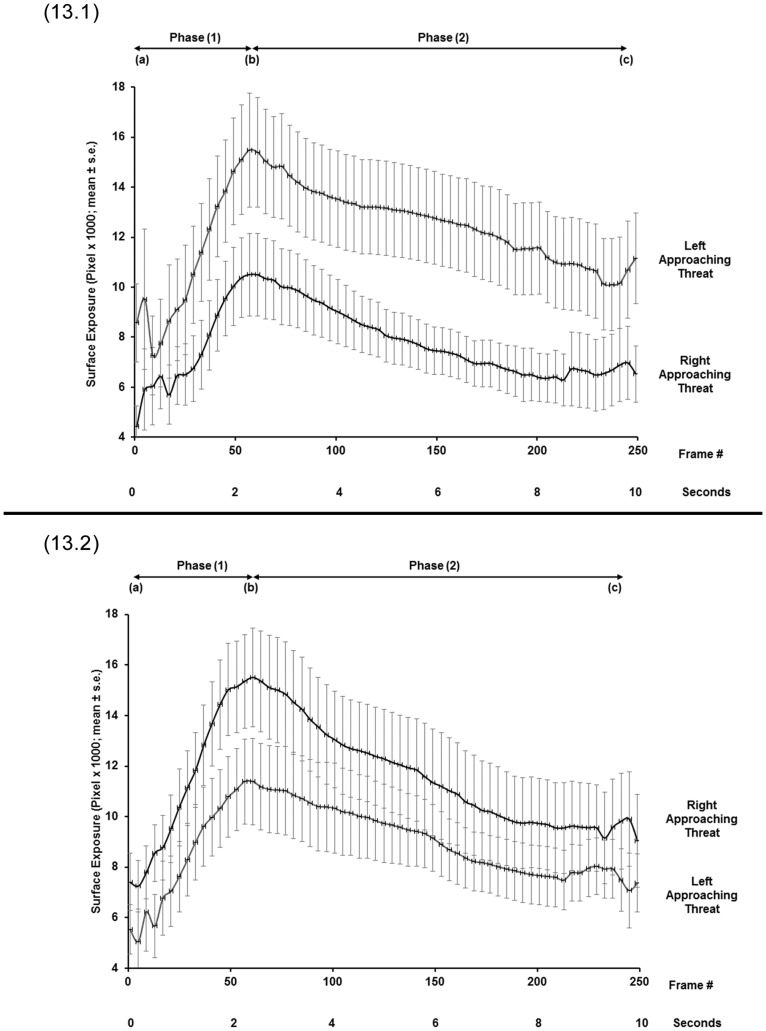
Avoidance response patterns of the two side-biased groups on a wide pole. Ventral surface exposure (mean ± SE) on wide poles in response to right- or left-approaching threats in the right-biased (13.1, N  = 10) and in left-biased (13.2, N  = 7) groups. Exposure readings are at 200-ms intervals, (A) at the onset of pole rotation, (B) at the end of pole rotation, and (C) at the end of the test.

**Figure 14:**
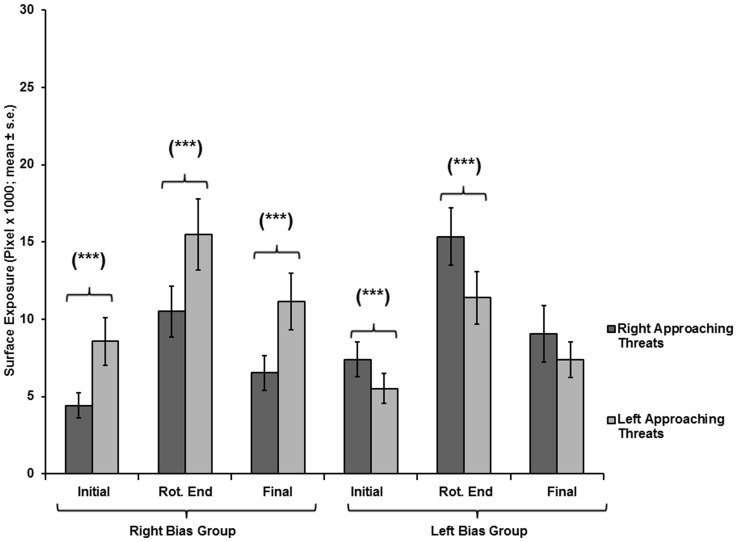
Ventral surface exposure of the two side-biased groups on a wide pole, as a function of threat-approach direction. Exposure (mean ± SE) under right- or left-approaching threats, for chameleons of the right-biased (N  = 10) and left-biased (N  = 7) groups, in tests on a wide pole at the onset of pole rotation (Initial), end of pole rotation (Rotation end), and end of test (Final).

### Tests on Narrow Poles

In the tests on narrow poles, for the right-biased group, all three spatial parameters were significantly lower for right-approaching threats than for left-approaching threats (Initial exposure: F_(1,13)_  = 16.721, p  = 0.001; End of rotation exposure: F_(1,13)_  = 18.049, p  = 0.001; Final exposure: F_(1,13)_  = 10.853, p  = 0.006) (Figs. 10.1, 11). For the left-biased group, all three spatial parameters were significantly lower in tests on left-approaching threats vs. right-approaching threats (Initial exposure: F_(1,10)_  = 10.465, p  = 0.009; End of rotation exposure: F_(1,10)_  = 13.051, p  = 0.005; Final exposure: F_(1,10)_  = 5.586, p  = 0.04) (Figs. 10.2, 11). The latency to final exposure (Fig. 12) did not differ between right- and left-approaching threats in either the right-biased group (F_(1,13)_  = 0.513, p  = 0.487) or the left-biased group (F_(1,10)_  = 0.086, p  = 0.775).

### Tests on Wide Poles

In tests on wide poles, for the right-biased group, all three spatial parameters were significantly lower in tests on right-approaching threats than on left-approaching threats (Initial exposure: F_(1,9)_  = 14.412, p  = 0.004; End of rotation exposure: F_(1,9)_  = 12.269, p  = 0.007; Final exposure: F_(1,9)_  = 17.058, p  = 0.003) (Figs. 13.1, 14). For the left-biased group, only the Initial exposure and the End of rotation exposure were significantly lower for left-approaching threats than for right-approaching threats (Initial exposure: F_(1,6)_  = 26.203, p  = 0.002; End of rotation exposure: F_(1,6)_  = 31.063, p  = 0.001) (Figs. 13.2, 14). The parameter of Final exposure did not differ between the right- and left-approaching threats (F_(1,6)_  = 2.386, p  = 0.173). The latency to final exposure (Fig. 12) also did not differ between the right- and left-approaching threats in either the right-biased group (F_(1,9)_  = 1.049, p  = 0.333) or the left-biased group (F_(1,6)_  = 0.217, p  = 0.658).

## Discussion

When a threat stimulus [Videos S2 and S3] is moved in an arc around a chameleon perched on a stationary pole, the chameleon will respond in a precise counter-rotation, keeping the pole between it and the threat. At the functional level, lower exposure to a threat, specifically the “Final exposure”, implies better concealment. With its surrounding obscured by an opaque screen and no obvious threat, no such position corrections are observed, leading to the conclusion that the avoidance response to threat is mediated by vision. The precise nature of the avoidance response closely resembles the previously described “station keeping” observed mainly in insects [1], [2], [6] For example, bees maintain their spatial positioning precisely relative to their hive’s entrance [2], [3], while water-striders (*Geris paludum* F.) do so in relation to water flow [4]. Using smooth pursuit, male blowflies (*Lucelia* spp.) view visual targets of interest by specific ommatidia [34]. Male hoverflies (*Syritta pipiens*) visually track and intercept flying females for copulation by maintaining the image of the female fixated on the frontal facets of both eyes [6]. This is performed with extreme accuracy by translating the angular position of the target on the retina to the angular velocity of the tracking fly, with response latencies of ca. 20 ms.

### Lateralization at the Population Level

At the population level, lateralization was observed in the Final exposure, with better concealment (i.e., lower exposure) when threats approached from the right, under both monocular and binocular viewing. No such lateralization was observed in the Initial exposure or in the End of rotation exposure. In comparison, eye use under these conditions [10] showed lateralization only when binocular viewing was possible. There is thus no obvious correspondence between lateralization of eye use and body use. This may support the view that lateralization here stems from the brain’s sensory-motor functions rather than merely from the information provided by the eyes.

### Anti-symmetry of Lateralization in the Population

That the chameleons were either “right biased” or “left biased”, as judged by their individual performance, points to the existence of two similar-sized sub-populations. Comparisons between responses to right- and left-approaching threats within each sub-population revealed significant differences in the three examined spatial parameters, but not in the temporal parameter (i.e., latency to final exposure). Each sub-population was lateralized with respect to a given threat-approach direction: individuals of the “right-biased” sub-population were better concealed from threats approaching from the right, while individuals of the “left-biased” sub-population were better concealed from threats approaching from the left. This bimodality of response is expressed in the exposure values (mean and SE) of any given sub-population to threats approaching from the right that do not overlap with the exposure values to threats approaching from the left. The divergence in response is depicted in Figs 10.1, 10.2 and 13.1. In contrast, the exposures of the left-biased group in response to right- and left-approaching threats (Fig. 13.2) did overlap in the values of the two threat-approach directions toward the end of the response (see further on). Since the sub-populations were of roughly similar proportions, the situation is regarded as “anti-symmetrical” [16]. It should be noted that such a bimodal distribution of side-biased individuals in a population is not as common as asymmetric distributions. Most populations tested for lateralization in terms of handedness, fleeing, strikes and other behavioral patterns are asymmetrically distributed, with a majority biased toward one side and a minority toward the other [13], [14], [16], [35], [36]. Anti-symmetrical distribution of lateralization has been demonstrated, for example, in rats (*Rattus norvegicus*) [37], with ca 10% of individuals in the population being ambidextrous and the remainder equally divided between right- and left-handed individuals. An octopus (*Octopus vulgaris*) hiding in an aquarium also displays anti-symmetry in eye use when viewing prey presented to it [38].

Here, the fact that the tested population is weakly lateralized (and only in the parameter of Final exposure), yet comprises sub-populations that are strongly lateralized (in all three parameters), makes sense in light of their relatively homogeneous natural habitats. In these arboreal habitats, the chances of confronting a threat from a given three-dimensional position are equal, and the chances of a threat confronting a left- or right-biased chameleon are also equal. This may be regarded as an evolutionary solution to preventing predators from using lateralization to their advantage.

A noticeable difference between the right- and the left-biased groups was observed with respect to final body exposure. The Final exposure values of the right-biased group were lower (i.e., better concealment) for right-approaching vs. left-approaching threats, for all pole widths. The Final exposure of the left-biased group on a wide pole was kept relatively low for both threat-approach directions. This, together with the similarity of exposure levels to right-approaching and left-approaching threats on a narrow pole, underlies the overall better concealment of the entire population when responding to right-approaching threats. Our results show that under monocular viewing (a wide pole), the left-biased group exerted a relatively similar and efficient avoidance response to threats from both sides, a feat not accomplished by the right-biased group. In other words, the performance of the left-biased group did not mirror that of the right-biased group. Under binocular viewing (on a narrow pole), the responses of each of the side-biased groups to threats from the right or left differed significantly, under all conditions. Comparably, in humans, left-handed individuals using their left hand perform better in given tasks than do right-handed individuals using their right hand [39]. Moreover, there is no difference between left-handed and right-handed individuals in performing ballistic tasks and visually guided tasks. However, for visually guided tasks, left-handed individuals perform better with their right hand compared with right-handed individuals using their left hand [40]. These examples demonstrate that given tasks executed by individuals of opposite handedness, with their preferred or non-preferred hand, do not result in the same level of performance.

An individual belonging to a given biased group in narrow pole tests could respond as belonging to the opposite bias group during wide pole tests. The population thus comprises individuals with a stable bias toward a given side and individuals with a transient bias, depending on whether the visual input is monocular (wide pole) or binocular (narrow pole).

The motor responses observed here were accomplished by the chameleon’s using the pole as the axis of rotation. Although there are numerous examples of lateralized limb use, we did not consider this as a lateralization factor. The chameleons’ responses were also analyzed in terms of eye use [10], showing lateralization as a function of monocular/binocular viewing (pole width) and direction of threat approach. We assume that this lateralization of eye use projects onto the observed motor responses.

Obvious disadvantages of lateralization will occur when, for example, the probability of encountering prey or predator is similar for both sides of the body. In that case, having one side of a sensory system less efficient in identifying or responding to stimuli will be deleterious to the organism’s survival [28], [41].

Many species, such as ground-dwelling birds and amphibians, show lateralization at the behavioral level and live in a world dominated by two distinct visual domains. One is the nearby surfaces, such as ground or water, where food is found and social interactions occur. The other is the above-head space, where avian predators are likely to appear. Thus, for the fiddler crab (e.g. *Uca vomeris*) living on mudflats, objects moving below the horizon are regarded as conspecifics while those above the horizon are considered predators [42]. Such a division of the visual world may have been an evolutionary force toward laterality, whereby each eye specialized in a given domain. In contrast, in dense foliage, prey or predators may appear abruptly at close range and in any spatial position, conditions under which laterality may be detrimental.

Lateralized motor patterns include pawdness in bufonids during body righting or removal of disturbances [24], [43], escape responses in lizards [44] and foot use in birds [45]–[48]. In contrast, lateralization in the chameleons’ avoidance response was found only when the motor patterns were analyzed at the finer individual and population levels, as well as spatio-temporal levels. Moreover, while certain components of the response (Initial exposure and End of rotation exposure) did show lateralization, other components (Final exposure) did not. In the Final exposure, laterality was observed in all responses except for the left-biased group under monocular viewing. Forcing behavior patterns into bimodal categories may therefore prevent us from seeing the more precise underlying picture.

## Supporting Information

Video S1 **A control experiment aimed to determine if chameleons correct their position using vestibular-driven compensatory responses.** The chameleons were tested on a rotating pole within an opaque sphere, providing no visual threat. Pole rotation was at an angular velocity of ∼15°/s.
(WMV)

Video S2 **A control experiment aimed to determine if chameleons correct their position using visual but not vestibular information.** The chameleons were tested on a stationary pole with a threat stimulus moved at an arc of ca. 80°, 50 cm from the pole. Threat angular velocities were 15°, 35° and 70°/s.
(WMV)

Video S3 **A demonstration of the position correction of a chameleon on a vertical pole, in response to a threat (a hand) moved at an arc around it.**
(WMV)
